# Single-cell landscape of immunological responses in elderly patients with sepsis

**DOI:** 10.1186/s12979-024-00446-z

**Published:** 2024-06-22

**Authors:** Wanxue He, Chen Yao, Kaifei Wang, Zhimei Duan, Shuo Wang, Lixin Xie

**Affiliations:** 1 Department of Pulmonary and Critical Care Medicine, Xuanwu Hospital Capital Medical University, Beijing, China; 2 College of Pulmonary and Critical Care Medicine, The Eighth Medical Center, Chinese PLA General Hospital, Beijing, China; 3 CAS Key Laboratory of Pathogen Microbiology and Immunology, Institute of Microbiology, Chinese Academy of Sciences, Beijing, China

**Keywords:** Sepsis, Aging, Immunosenescence, immunopathogenic mechanisms, Single-cell sequencing, Immune dysfunction

## Abstract

Sepsis is a dysregulated host response to severe infections, and immune dysfunction plays a crucial role in its pathogenesis. Elderly patients, a special population influenced by immunosenescence, are more susceptible to sepsis and have a worse prognosis. However, the immunopathogenic mechanisms underlying sepsis in elderly patients remain unclear. Here, we performed single-cell RNA sequencing of peripheral blood samples from young and old subjects and patients with sepsis. By exploring the transcriptional profiles of immune cells, we analyzed immune cell compositions, phenotype shifts, expression heterogeneities, and intercellular communication. In elderly patients with sepsis, innate immune cells (e.g., monocytes and DCs) exhibit decreased antigen presentation, presenting an overactive inflammatory and senescent phenotype. However, the immunophenotype of T cells shifted to characterize effector, memory, and exhaustion. Moreover, we identified strong interferon-γ responses of T cells in both aging and sepsis groups and a deranged inflammaging status in elderly sepsis patients. Tregs in elderly patients with sepsis showed increased abundance and enhanced immunosuppressive effects. In addition, metabolism-associated pathways were upregulated in T cells in elderly patients with sepsis, and the lysine metabolism pathway was enriched in Tregs. Cell–cell interaction analysis showed that the expression profile of ligand-receptor pairs was probably associated with aggravated immune dysfunction in elderly patients with sepsis. A novel HLA-KIR interaction was observed between Tregs and CD8 + T cells. These findings illustrate the immunological hallmarks of sepsis in elderly patients, and highlight that immunosuppressive and metabolic regulatory pathways may undergo important alterations in elderly patients with sepsis.

**Supplementary Information:**

The online version contains supplementary material available at 10.1186/s12979-024-00446-z.

## Introduction

Sepsis is a life-threatening syndrome caused by dysregulated host responses to severe infections, and leads to high morbidity and mortality rates in intensive care units worldwide [1]. Remarkably, elderly individuals display increased susceptibility to sepsis, which progresses much more rapidly to septic shock, multiple organ dysfunction, and other serious complications, thereby leading to a worse prognosis. As reported by Martin et al., the relative risk of sepsis is 13.1 times higher in elderly patients than in adults [2]. Moreover, the average 30-day mortality rate for sepsis has been estimated to be 24.4% worldwide over the last decade [3]. The average mortality rate from sepsis in the elderly increased by at least 10% than that in young patients [2]. As the global population ages, the morbidity and mortality of sepsis in the elderly will likely continue to increase and greatly aggravate the global economic and medical burden. Despite the magnitude of this issue, the pathogenesis of sepsis in the elderly is not well understood.

Although an increasing number of studies aimed at looking for therapeutic approaches to reduce sepsis-related mortality, sepsis still accounts for approximately 20% of deaths each year [3]. This proportion is undeniably higher among elderly patients. As sensitive biomarkers or effective and specific therapeutic agents remain lacking, there is an urgent need to better understand the pathophysiological mechanisms in elderly patients with sepsis, with particular focus on the immunopathogenic mechanisms.

Immune dysfunction plays a crucial role in poor prognosis of sepsis [4]. It is widely accepted that overactive immune responses leading to organ dysfunction, immunosuppression, or immune exhaustion in the late period of sepsis can directly and/or indirectly contribute to poor outcomes in patients [5]. Furthermore, elderly patients are more susceptible to severe infectious diseases and have a poorer prognosis, at least partly because of the deterioration of the immune system caused by aging, which has been termed as “immunosenescence” or “inflammaging” [6]. Therefore, the immunopathology of sepsis and aging has received considerable attention to better understand the influence of immune dysregulation on both sepsis and aging. High-throughput technological advances have made it possible to detect unbiased signatures of the immune system at the single-cell level. Using single-cell sequencing, Yao et al. reported that cDCs exhibit an immunoregulatory phenotype in septic mice associated with hyperinflammation and organ dysfunction [7]. Moreover, recent research on patients with sepsis showed that the interferon response was upregulated and T/NK cells presented both exhausted and activated properties [8]. Several studies have explored age-associated alterations in the immune cells. Aging shifts the polarization of T cells from naïve and memory phenotypes to effector, exhausted, and regulatory phenotypes. In addition, inflammatory macrophages and dysfunctional DCs are increased in aging patients, thereby aggravating senescence-associated pro-inflammatory endotypes and immune dysfunction [9]. These observations revealed the heterogeneity of various immune cell subtypes underlying sepsis and aging. In elderly patients with sepsis, underlying alterations in the immune system are largely responsible for the increased morbidity and mortality. Although many unique immunological phenotypes have been reported in aging and sepsis patients respectively, a comprehensive landscape of immune cell alterations, including immune cell composition, expression heterogeneity and cellular interaction, in elderly patients with sepsis is still lacking.

Here, we used single-cell transcriptional sequencing to comprehensively explore the heterogeneity of peripheral blood mononuclear cells (PBMCs) in elderly patients with sepsis at the single-cell level. This study provides an unbiased landscape of immune cell alterations, including immune cell compositions, expression heterogeneities, and cellular interactions in elderly patients with sepsis, which will facilitate a better understanding of the critical nodes between the dysregulated immune system and severe infections that occurs in elderly patients, and may also shed light on possible novel therapeutic strategies.

## Methods

### Ethnics statement

The ethical consent of this study was approved by the Ethics Committee of Chinese PLA General Hospital (Beijing, China).

## Patient cohort and clinical characteristics

Twelve samples included patients and controls in this study were enrolled from the First Medical Center of PLA General Hospital and enrolled in the study from 16 April to 30 June 2020. The demographic characteristics of the 12 samples studied by scRNA-seq and scTCR-seq were listed in Supplementary Table 1, which included three young healthy adults (Control) (29–45 years old), three elderly non-sepsis patients (E-NoSEP) (≥ 80 years old), three young sepsis patients (Y-SEP) (46–57 years old), and three elderly sepsis patients (E-SEP) (≥ 80 years old). And all clinical data were collected from the sampling day.

As a significant heterogeneous disease, manifestations of sepsis vary with etiological factors, which may make it much more difficult to explore the underlying immunopathology. Because pneumonia is one of the most common primary causes for sepsis clinically [8], sepsis patients enrolled in this study were individuals diagnosed as sepsis secondary to pneumonia.

The inclusion criterions of enrolled sepsis patients were as following: age ≥ 18 years; clinical diagnosis of sepsis as defined by the Third International Consensus Definitions for Sepsis in 2016 (Sepsis-3) [10]; the sepsis was secondary to pneumonia. While the exclusion criterions consisted of: diagnosed as malignancy or autoimmune diseases; use of chronic corticosteroids or immunosuppressive agents within 30 days; pregnancy.

## PBMC isolation

Peripheral blood samples were collected from patients who were enrolled in this study. For sepsis patients, blood samples were obtained within 24 h as have been diagnosed. PBMC was prepared with density-gradient centrifugation. Cell viability was verified with trypan blue. For each sample, cell viability exceeded 90%. Then followed by single-cell RNA sequencing.

## Single-cell RNA sequencing and quality control

The prepared cell suspensions were subjected to Single Cell 5′ Library and Gel Bead kits (10X Genomics) and Single Cell V(D)J Enrichment kits (10X Genomics) according to the manufacturer’s protocol. Briefly, the cell suspension was loaded onto a Chromium controller (10X Genomics) to generate barcoded gel beads in GEMs. Followed by reverse transcription inside each GEMs and then cDNA along with cell barcode identifiers was amplified, after which, sequencing libraries were prepared and normalized on Illumina platform [11]. The data was aligned to the GRCh38 reference genome using CellRanger (10X Genomics). The output filtered gene expression matrices were selected for further analyses only if met the following criteria: gene number > 200, unique molecular identifier (UMI) count > 800, and the percentage of mitochondrial gene < 10% [12].

### Dimensionality reduction and clustering

After filtering, a total of 105,216 cells with high quality were analyzed by Seurat R package (v.3.0.0) [13]. Gene expression matrices were normalized and top 2,000 highly variable features were obtained. The integrated data was subjected to scale and principal-component analysis (PCA) were performed to reduce nonlinear dimensionality. And then we clustered or sub-clustered cells using the K-Nearest Neighbour and FindClusters functions. The UMAP was used to visualize the results of clustering or sub-clustering according to the expression levels of canonical markers of particular cell types and subtypes [12].

### DEG identification and functional enrichment analysis

Differential gene expression analysis was performed using the FindMarkers function in Seurat R packages with default parameters [11]. DEGs were filtered using a minimum log2 (fold change) of 0.25 and a maximum FDR value of 0.01.

For functional enrichment analysis of DEGs, GSEA, GO, and KEGG enrichment were performed using clusterProfiler packages. Hallmark gene sets in MSigDB were used for annotation [14].

### Defining cell state scores

We used cell scores to evaluate the average expression level of the genes from a certain predefined gene set in the respective cells. The scores were calculated as the difference between the average expression levels of each gene set. Specifically, APOPTOSIS SIGNALING PATHWAY (GO:0097190), 12 cytotoxicity-associated genes (PRF1, IFNG, GNLY, NKG7, GZMB, GZMA, GZMH, KLRK1, KLRB1, KLRD1, CTSW and CST7), and 6 well-defined exhaustion markers (LAG3, TIGIT, PDCD1, CTLA4, HAVCR2 and TOX) were used to define apoptosis, cytotoxicity and exhaustion score, respectively [12].

### TCR V(D)J sequencing and analysis

Full-length TCR V(D)J segments were enriched using Chromium Single-Cell V(D)J Enrichment kits (10X Genomics) according to the manufacturer’s protocol. Only cells with at least one productive TCR α- and β-chain were kept for further analysis. Cell Ranger vdj pipeline (v.3.0.2) was performed to achieve TCR clonotype assignment [12].

### Trajectory and pseudotime analysis

Trajectory and pseudotime analysis were performed using monocle version 2.14.0. Raw count data was converted to a CellDataSet object using the import CDS function in monocle. Genes that expressed differentially between subsets were set as the ordering genes. The minimum spanning tree was constructed using the reduceDimensions function [15].

### Cell–cell communication analysis

We applied the Cellphone database of known receptor-ligand pairs to assess cell–cell communication. Genes from the Seurat object were reformatted into the input format described on the CellphoneDB website. Cells were fed into cellphonedb calculation program using 50 iterations, precision of 3, and 0.1 ratio of cells in a cluster expressing a gene. Then interactions trimmed based on significant sites with *p* < 0.05.

### Statistics

The statistical methods and threshold for each analysis were described in the figure legends or Methods sections.

## Results

### Single-cell transcriptional profiling of immune cells in elderly patients with sepsis 

**Fig. 1:**
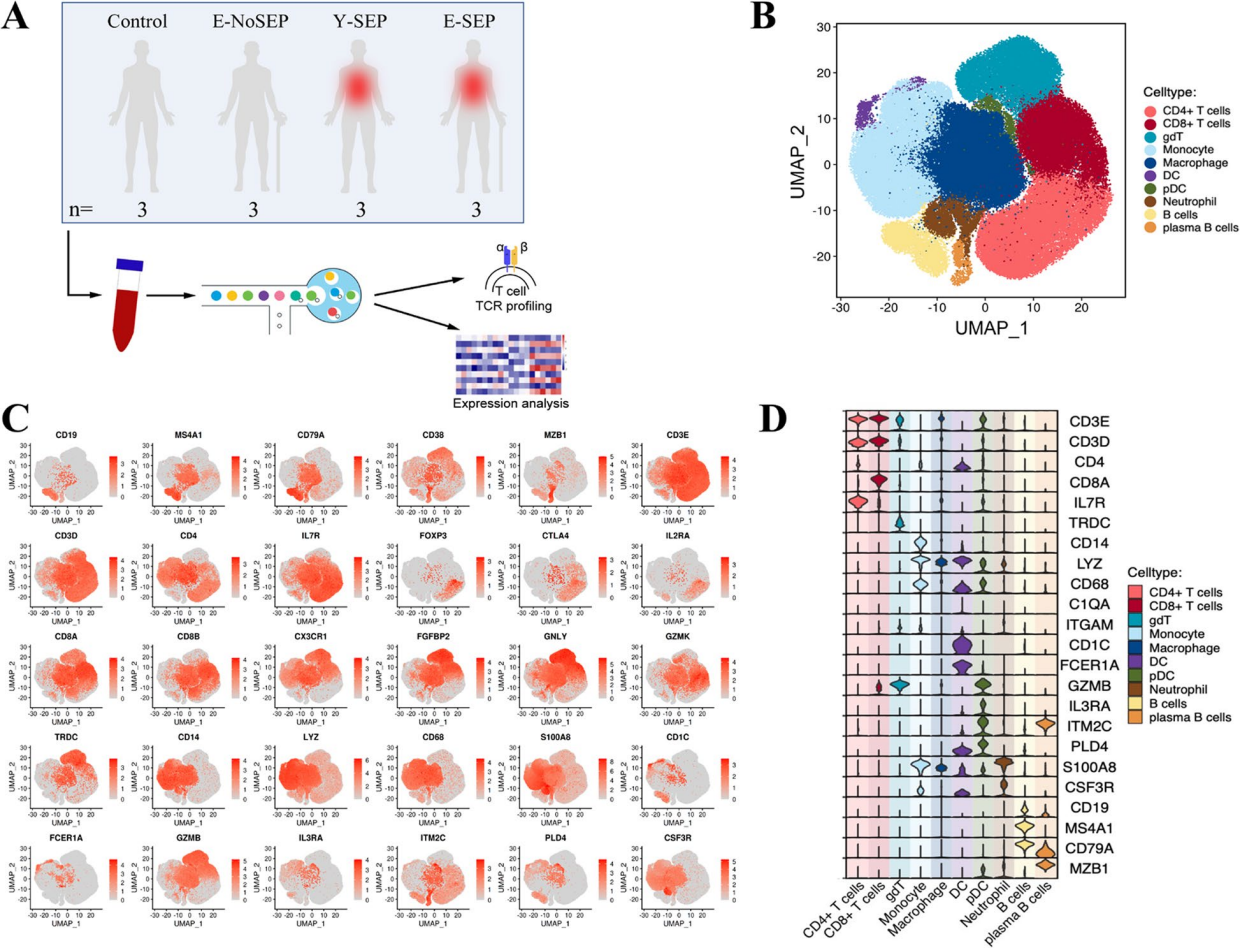
Study design and single-cell transcriptional profiling of PBMCs from controls and patients. **A**, An overview of the study design and workflow. The scRNA-seq was applied to PBMCs across four conditions, including young healthy adults (Control), elderly non-sepsis patients (E-NoSEP), young sepsis patients (Y-SEP), elderly sepsis patients (E-SEP), and the output data was used for expression analysis and TCR profiling. **B**, Visualization of major indentified cell types. The UMAP embedding of 105216 single cells from Control (*n*=3), E-NoSEP (*n*=3), Y-SEP (*n*=3), E-SEP (*n*=3) samples, showing the formation of 10 clusters (cells for CD4+T, CD8+T, γδT, monocyte, macrophage, DC, pDC, neutrophil, B, and plasma B, respectively). Each dot corresponds to a single cell, colored according to cell type. **C**, The UMAP plot showed the expression of canonical cell markers used to label each cluster. Data are colored according to expression levels. **D**, The scaled expression distribution of selected canonical cell marker genes in the identified 10 clusters, shown by Violin plots

To characterize the immunological features of elderly patients with sepsis at the single-cell level, we performed scRNA-seq (10X Genomics) to study the transcriptional profiles of PBMCs across four groups: young healthy adults (Control) (*n* = 3), elderly non-septic patients (E-NoSEP) (*n* = 3), and young sepsis patients (Y-SEP) (*n* = 3), elderly sepsis patients (E-SEP) (*n* = 3). A schematic representation of the study design and workflow is shown in ​Fig. 1A. The clinical characteristics of the enrolled patients are presented in Supplementary Table 1. After filtering and quality control (see Methods), we performed further analyses on 105, 216 cells (Fig. 1C). A UMAP plot was used to visualize the results of dimensionality reduction and clustering (Fig. 1B, Fig. S1A). We identified erythroid (*HBB*), cycling cell (*MKI67*), platelet (*PPBP*, *TUBB1*, *PF4*), stress cell (*HSPB1*, *DNAJB6*, *HSPH1*, *GADD45B*), and 10 major immune cell clusters, including CD4 + T (*CD3*, *CD4*, *IL7R*), CD8 + T (*CD3*, *CD8*), γδT (*TRDC*), monocyte (*CD14*, *LYZ*, *CD68*), macrophage (*LYZ*), DC (referring to cDC) (*CD1C*), pDC (*IL3RA*), neutrophil (*CSF3R*), B (*CD19*, *MS4A1*, *CD79A*), and plasma B (*MZB1*) (Fig. 1, Fig. S1). The clusters were annotated according to the expression of canonical specific markers, and the scaled expression distributions of the selected signature genes are shown in Fig. 1C-D and Fig. S1B. The annotation of cell clusters in the UMAP plots was comparable among the four groups (Fig. S1C), which indicated that the cell type identity was not altered by sepsis or aging.

### Alterations in cell compositions associated with aging and sepsis 

**Fig. 2:**
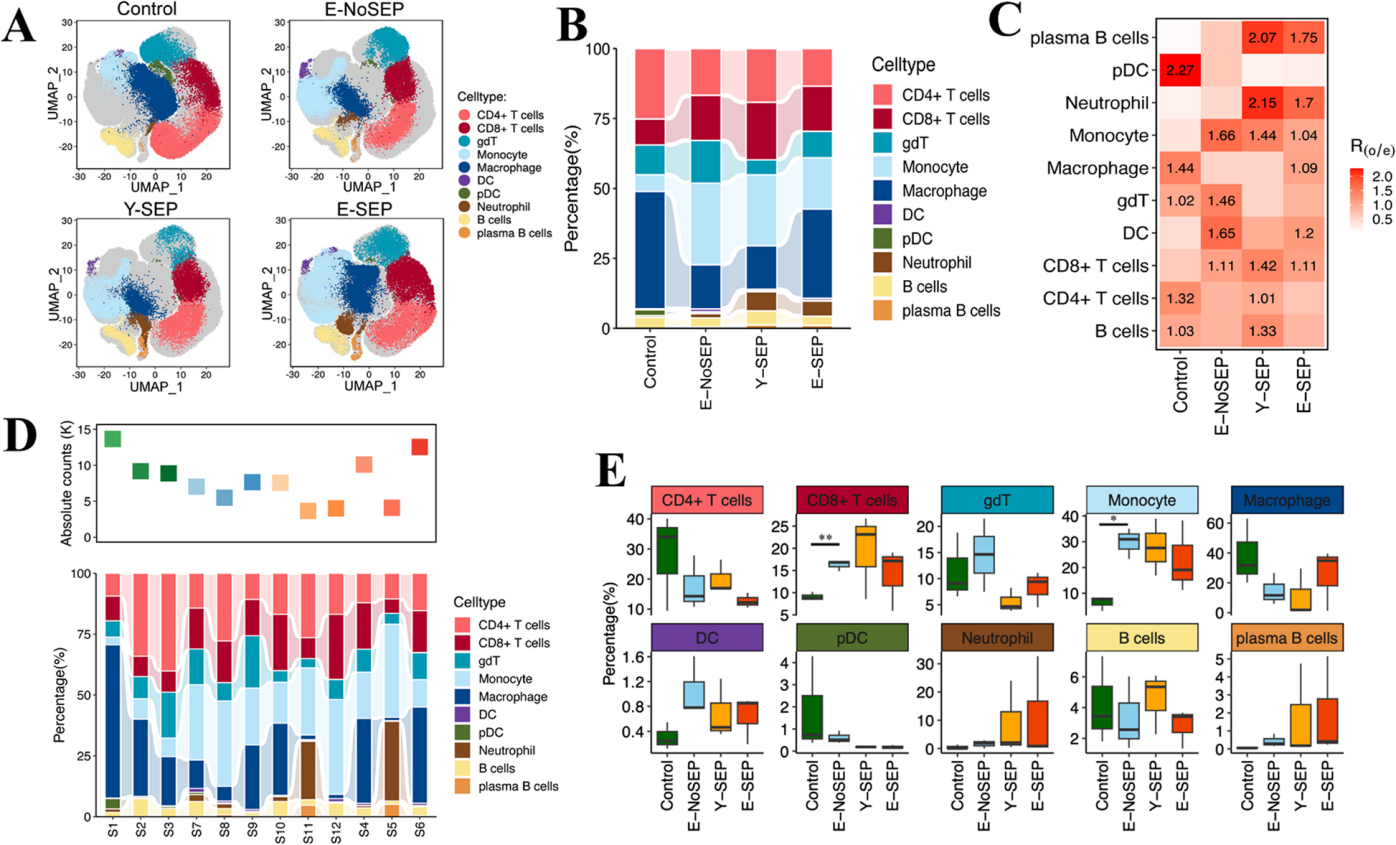
Differences in cell compositions across four conditions. **A**, UMAP projection of the Control, E-NoSEP, Y-SEP, E-SEP conditions. Each dot corresponds to a single cell, colored according to cell type.  **B**, The bar plot shows the average proportion of each cell type derived from different groups of patients.  **C**, Heatmap represents cluster preference across four conditions estimated by Ro/e score. One cluster is identified as being enriched in a condition if Ro/e>1. **D**, The top dot plot shows the sum of absolute counts of lymphocytes and monocytes in the PBMCs of each sample. The bottom bar plot shows the relative contribution of each cluster in each studied subject. **E**, The percentage of major immune cell types across four conditions. Conditions are shown in different colors. Horizontal lines represent median values, with whiskers extending to the furthest data point within a maximum of 1.5 × interquartile range. All differences with *P* < 0.05 are indicated. *P < 0.05, **P < 0.01; T-test was used for analysis

To reveal the impact of sepsis and aging on immune cell composition, we compared the proportions of 10 major cell types in PBMCs across the four groups (Control, E-NoSEP, Y-SEP, E-SEP) (Fig. 2). Cells from each group are shown as separate UMAP plots in Fig. 2A, whereas the proportion and cluster preference across the four groups and 12 individuals are shown in Fig. 2B-E. We found that the proportion of CD4 + T cells decreased in both aging and septic states by comparing E-NoSEP to Control and Y-SEP to Control separately (Fig. 2B-E), which is consistent with previous reports of aging-induced decreased expansion and sepsis-induced increased apoptosis in CD4 + T cells [16]. Remarkably, we observed a trend of decreased CD4 + T cell percentages in E-SEP compared to Y-SEP, as shown in Fig. 2E. Therefore, the decrease in CD4 + T cells was amplified in elderly patients with sepsis, which might indicate that CD4 + T cells are more susceptible to apoptosis and that proliferation is further impaired. In contrast, the proportion of CD8 + T cells increased significantly in the E-NoSEP group compared to that in the Control group (Fig. 2E). B cells analysis revealed that the proportion of B cells decreased in both the E-NoSEP and E-SEP groups, whereas plasma B cells increased in the E-SEP and Y-SEP groups (Fig. 2B-E). The increase in plasma B cells in the sepsis group was in accordance with a study by Wang et al. [8].

Our data also showed differences in the composition of innate immune cells. We found that the percentage of neutrophils increased in both the E-SEP and Y-SEP groups (Fig. 2B-E), which was parallel to the general expansion of neutrophils during infection. Additionally, the proportion of monocytes increased significantly in the E-NoSEP group compared to that in the Control (Fig. 2E), which is consistent with previous reports by Zheng et al. [9]. Conversely, the number of macrophages decreased in both the E-NoSEP and Y-SEP groups compared to the Control (Fig. 2B-E). We also found that the percentage of cDCs increased, whereas that of pDC decreased with aging and sepsis (Fig. 2B-E). In summary, these results demonstrated that the composition of immune cells differs in both aging and sepsis states.

### Decreased antigen presentation, increased inflammaging and apoptosis phenotype of innate immune cells were observed in elderly sepsis patients

**Fig. 3:**
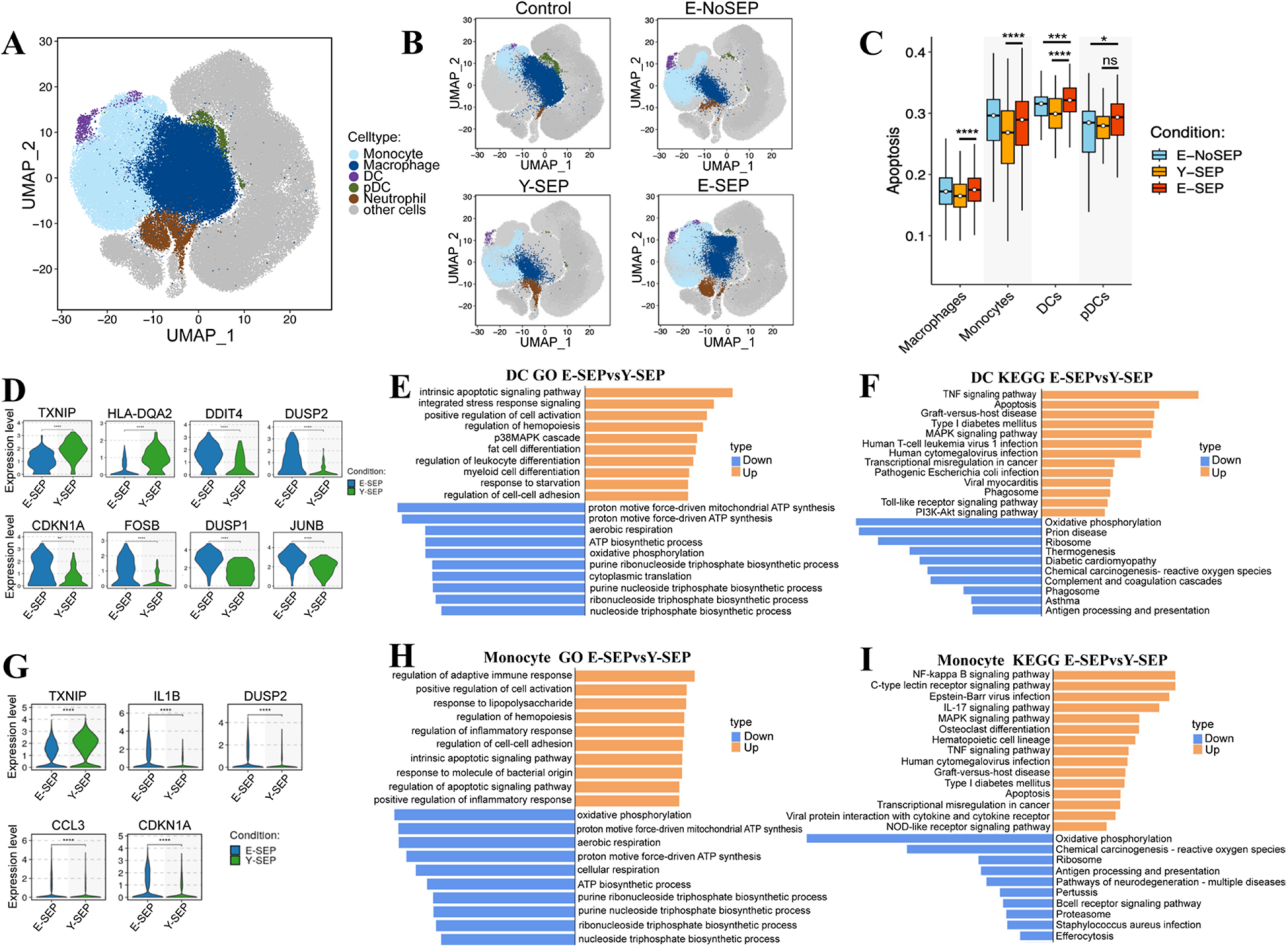
Characterization of innate immune cell subsets across four conditions. **A**, UMAP projection of innate immune cells, including monocytes, macrophages, DCs (conventional DCs), pDCs (plasmacytoid DCs), and neutrophils. Each dot corresponds to a single cell, colored according to cell type.  **B**, UMAP projection of innate immune cells among Control, E-NoSEP, Y-SEP, E-SEP conditions.  **C**, Box plots of the apoptosis score across different cell types and conditions. Conditions are shown in different colors. Horizontal lines represent median values, with whiskers extending to the furthest data point within a maximum of 1.5 × interquartile range. All differences with *P* < 0.05 are indicated. *P < 0.05, ***P < 0.001, ****P < 0.0001; unpaired T-test was used for analysis. **D**, Violin plots showing the distribution of normalized expression levels of selected antigen-presenting, inflammation and aging-associated genes in DC cluster between E-SEP and Y-SEP groups. All differences with *P* < 0.05 are indicated. **P < 0.01, ****P < 0.0001; T-test was used for analysis. **E**, Representative GO-BP terms enriched in the top 100 upregulated or downregulated DEGs based on functional enrichment analysis in DCs between E-SEP and Y-SEP groups. GO terms are labeled with name, and sorted by −log_10_ (*P*) value. The top 10 enriched GO terms are shown. **F**, The KEGG pathway enrichment analysis of the top 100 upregulated or downregulated DEGs in DCs between E-SEP and Y-SEP groups. Selected pathways are shown and labeled with name, and sorted by −log_10_ (*P*) value. **G**, Violin plot similar to **D**, but for monocytes. **H**, GO-BP enrichment analysis similar to **E**, but for monocytes. **I**, The KEGG pathway enrichment analysis similar to **F**, but for monocytes

To further explore transcriptional alterations in innate immune cells in elderly patients with sepsis, we performed an integrated comparative analysis of differentially expressed genes (DEGs) in innate immune cell types, mainly between the E-SEP and Y-SEP groups (Fig. 3, Fig. S2). Cell subtype-specific gene signatures were analyzed by Gene Ontology (GO) and Kyoto Encyclopedia of Genes and Genomes (KEGG) enrichment, as well as by defining cell state scores.

By analyzing the DEGs in DCs between the E-SEP and Y-SEP groups, we found that DCs showed heterogeneous alterations (Fig. S2A). Among the upregulated DEGs in E-SEP compared to Y-SEP, we observed inflammation-associated genes, such as *FOSB*, *DUSP1*, and *JUNB*, and aging-associated genes, such as IFN-stimulated genes *DDIT4* and *DUSP2* (Fig. 3D), thereby indicating an overactive inflammatory response of cDCs in elderly patients with sepsis. Furthermore, *CDKN1A*, a well-known senescence hallmark gene, was significantly upregulated in the E-SEP group (Fig. 3D). Remarkably, *TXNIP* and *HLA-DQA2,* which play crucial roles in presenting antigens, were decreased in the cDCs of the E-SEP group (Fig. 3D). This suggests that the antigen-presenting ability of cDCs is impaired in elderly sepsis patients. By evaluating the GO biological process, significantly upregulated genes in the E-SEP group were enriched in the pathways of apoptosis, positive regulation of cell activation, and p38MAPK cascade, while downregulated genes were involved in mitochondrial ATP synthesis and oxidative phosphorylation (Fig. 3E). In addition, using KEGG enrichment analysis, we found that TNF signaling, apoptosis, and MAPK signaling pathways were significantly enriched in E-SEP. In contrast, oxidative phosphorylation and ribosome and antigen processing and presentation pathways were significantly downregulated in elderly patients with sepsis (Fig. 3F), which was highly consistent with the DEG expression. Similar to cDCs, the inflammatory, senescence, and apoptosis pathways were upregulated, whereas the antigen processing and presentation pathway was downregulated in pDCs in E-SEP (Fig. S2C, D and E). These results indicate that DCs exhibited decreased antigen-presenting ability and turned into an overactive inflammatory and aging phenotype in elderly patients with sepsis, which might be due to the synergistic effects of both aging and sepsis.

Next, we studied the expression patterns of the DEGs in both monocytes and macrophages. For monocytes, *TXNIP* was significantly downregulated in E-SEP compared to that in Y-SEP (Fig. 3G), which suggests impaired antigen presentation. In addition, aging-associated genes, such as the IFN-stimulated genes *IL1B*, *DUSP2*, and *CDKN1A*, and chemokines such as *CCL3,* were significantly upregulated in E-SEP compared to Y-SEP (Fig. 3G). Functional analysis revealed that the downregulated genes in E-SEP were involved in mitochondria-associated GO pathways, whereas the upregulated genes were enriched in the regulation of inflammatory responses and apoptotic signaling pathways (Fig. 3H). Consistently, inflammation-associated pathways, such as NF-kB, MAPK, and TNF signals, as well as apoptosis pathways were enhanced in monocytes from E-SEP, while oxidative phosphorylation, ribosome, and antigen processing and presentation pathways were inhibited (Fig. 3I). Similarly, inflammation-associated and apoptosis pathways for macrophages were enriched in E-SEP compared to Y-SEP (Fig. S2F-H). These alterations in monocytes and macrophages may provide clues to illustrate the phenomenon of endotoxin tolerance [17] driven by both sepsis and aging.

For neutrophils, we observed that the neutrophil extracellular trap formation pathway was significantly enhanced in E-SEPs (Fig. S2J). Although the formation of neutrophil extracellular traps (NETs) is generally beneficial for eliminating various pathogens [18], it has been reported that the over-interaction of NETs with platelets, complement, and the endothelium can promote the formation of immunothrombosis, which damages the microcirculation in sepsis [19]. We found that both complement and coagulation cascades and platelet activation pathways were significantly increased in the neutrophils of the E-SEP group (Fig. S2J), suggesting that the microcirculation might be damaged.

Given our observation that both the ribosome and apoptotic pathways changed in almost all innate immune cell types, we evaluated the expression levels of cellular apoptosis by defining cell state scores. We found that the apoptosis score was uniformly and significantly upregulated in all innate immune cells, including cDCs, pDCs, monocytes, macrophages, and neutrophils, in E-SEPs compared to that in Y-SEPs (Fig. 3C and Fig. S2K). This suggests that the innate immune cells are more susceptible to apoptosis in elderly patients with sepsis.

### Phenotype and function alterations of T cells in elderly patients with sepsis 

**Fig. 4:**
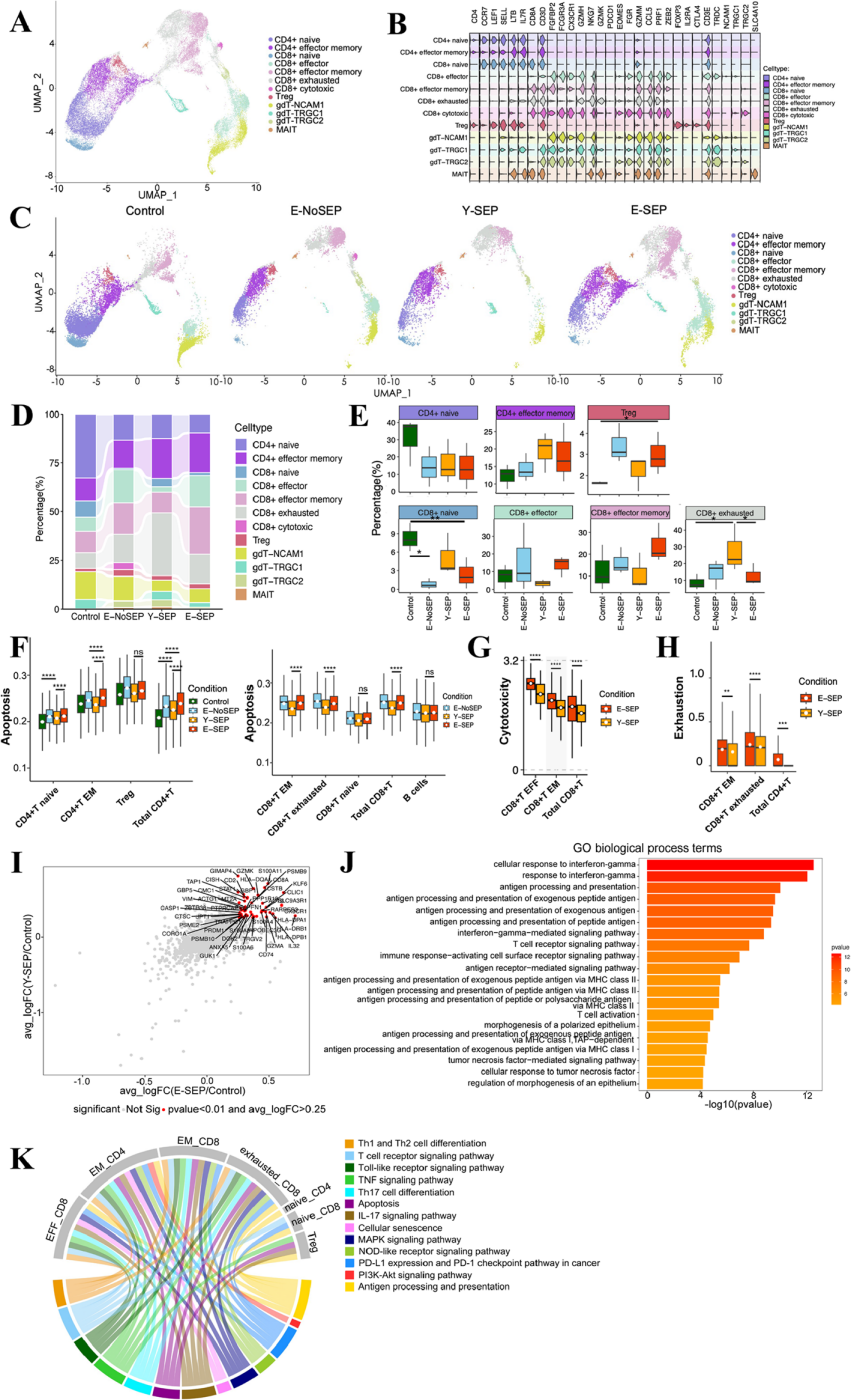
Immunological features of T cells. **A**, Sub-clustering of T cells. Each dot corresponds to a single cell, colored according to cell type. **B**, The expression distribution of canonical cell marker genes in the identified 12 T cell subsets, shown by Violin plots. **C**, UMAP projection of T cell subsets among Control, E-NoSEP, Y-SEP, E-SEP conditions. **D**, The bar plot shows the average proportion of each T cell subset derived from four conditions. **E**, The percentage of 7 identified T cell subsets across four conditions. Cytotoxic CD8+T cells were excluded in the following analysis. Conditions are shown in different colors. Horizontal lines represent median values, with whiskers extending to the furthest data point within a maximum of 1.5 × interquartile range. All differences with *P* < 0.05 are indicated. *P < 0.05, **P < 0.01; T-test was used for analysis. **F**, Box plots of the apoptosis score across different clusters and conditions. Conditions are shown in different colors. Horizontal lines represent median values, with whiskers extending to the furthest data point within a maximum of 1.5 × interquartile range. All differences with *P* < 0.05 are indicated. ****P < 0.0001; T-test was used for analysis. **G**, Box plots of the cytotoxicity score similar to **F**. ****P < 0.0001; T-test was used for analysis. **H**, Box plots of the exhaustion score similar to **F**. **P < 0.01, ***P < 0.001, ****P < 0.0001; T-test was used for analysis. **I**, Scatter-plot showing 49 DEGs in T cells of Y-SEP (*n* = 3) or E-SEP patients (*n* = 3) in comparison with Control (*n* = 3). Each red dot represents an individual gene with Wilcox test adjusted *P* value ≤0.01 and average log2 fold change ≥0.25 in the Y-SEP/Control and E-SEP/Control comparisons. **J**, Representative GO-BP terms enriched in the DEGs which are colored in red as in **I**. A redder one indicates a bigger* P* value. The top 20 enriched GO terms are shown. **K**, Circos plot showing the selected enriched KEGG pathway in DEGs of T cells between E-SEP and Y-SEP. The top chord widths represent different T cell subsets. The bottom chord widths represent different KEGG pathways which are shown in different colors

Both aging and sepsis have significant effects on T cells [16]. To characterize alterations of T cells across four groups, we subclustered total T cells and identified the following 12 subsets: 3 subtypes of CD4 + T cells (CD3 + CD4 +), 5 subtypes of CD8 + T cells (CD3 + CD8 +), 3 subtypes of γδT cells (CD3 + CD4-CD8-TRDC +), and MAIT (CD3 + SLC4A10 +). In CD4 + T cells, we defined CD4 + T naïve (*CCR7*, *SELL*), CD4 + T effector memory (CD4 + T EM) (*LTB*, *IL7R*) and regulatory T (Treg) cell (*FOXP3*, *IL2RA*) subtypes. Of the five subclusters of CD8 + T cells, we identified CD8 + T naïve (*CCR7*, *SELL*), CD8 + T effector (CD8 + T EFF) (*PRF1*, *NKG7*, *FGFBP2*), CD8 + T effector memory (CD8 + T EM) (*GZMH*, *NKG7*), CD8 + T cytotoxic (*FGR*, *GZMM*, *ZEB2*), and CD8 + T exhausted (CD8 + T EX) (*PDCD1*) subtypes (Fig. 4A, B, and Fig. S3A-B). It is noteworthy that the CD8 + T cytotoxic cluster was identified only in the S7 individual, thus, they were excluded from subsequent analysis.

To explore the specific phenotypic alterations in T cell subsets, we evaluated the distribution of each subcluster and found that the composition of T cell subsets differed across the four groups (Fig. 4C-E, and Fig. S3C-E). We observed that the proportion of naïve T cell subsets, including naïve CD4 + T and naïve CD8 + T subsets, decreased in E-NoSEP, E-SEP, and Y-SEP compared with that in the Control (Fig. 4D, E). In contrast, the proportion of active-state T cell subtypes, including CD4 + T EM, CD8 + T EFF, and CD8 + T EM, increased in both the E-NoSEP and E-SEP groups compared with that in the Control group (Fig. 4D, E). Remarkably, the CD8 + T EX subset increased in both E-SEP and Y-SEP, and a significant difference was observed in Y-SEP compared to that in the Control (Fig. 4D, E). In addition, the number of Treg subsets was significantly higher in the E-SEP group than in the Control group (Fig. 4E). These results demonstrate that both sepsis and aging induced an immune phenotypic shift to effector, memory, and exhausted populations.

We then evaluated the apoptosis, cytotoxicity, and exhaustion scores of the T cell subsets (Fig. 4F-H). With the exception of Treg and naïve CD8 + T cell subsets, the majority of T cell subsets showed significantly increased apoptosis scores in E-SEP compared to those in Y-SEP (Fig. 4F), thereby suggesting that T cells in elderly sepsis patients are more prone to apoptosis, which may contribute to more severe lymphopenia. Meanwhile, CD8 + T EFF, CD8 + T EM, and total CD8 + T cells showed higher cytotoxicity scores in E-SEP compared to Y-SEP (Fig. 4G). In addition, the exhaustion score was significantly upregulated in CD8 + T EX, CD8 + T EM, and total CD4 + T cells in the E-SEPs (Fig. 4H). Moreover, the cell state scores were in accordance with the phenotypic alterations of T cell subsets.

To gain further insight into the transcriptional characteristics of T cells, we performed an integrated analysis of DEGs and functional enrichment, and compared the effects of sepsis and aging, respectively. By estimating the upregulated genes between the E-SEP and Control groups and between the Y-SEP and Control groups, we found that interferon-γ-associated signal pathway was enriched (Fig. 4I-J), which were closely related to sepsis. Next, we analyzed the upregulated genes between the E-SEP and Control groups and between the E-NoSEP and Control groups to explore the effects of aging. Similarly, upregulated genes were also involved in interferon-γ-associated signal pathway (Fig. S3F-G). We then performed KEGG enrichment analysis of the upregulated genes in E-SEPs compared to Y-SEPs across the T cell subtypes (Fig. 4K). We observed that the upregulated genes were enriched mainly in classic signaling pathways related to sepsis, including apoptosis, MAPK, PD-1, PI3K-Akt, and TNF signaling pathways. In addition, the cellular senescence pathway was enriched in both the CD4 + T EM and CD8 + T EFF subsets of E-SEP. These results suggested that the characteristic of sepsis was much more remarkable, while the senescent feature was also observed in the elderly with sepsis, in which interferon-γ-associated signal may play an essential role.

### T-cell repertoire diversity and clonality in elderly patients with sepsis 

**Fig. 5:**
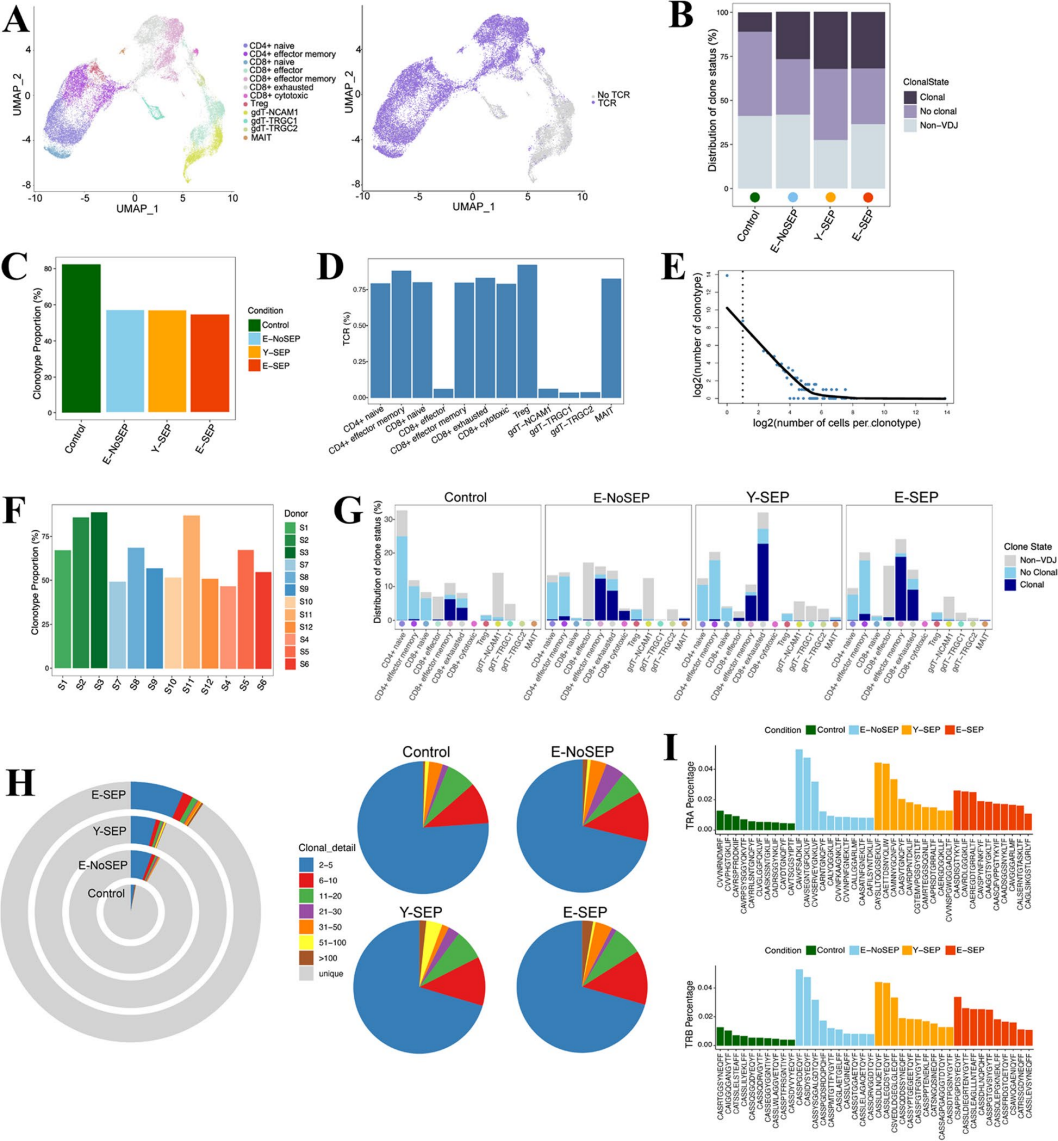
Expanded TCR clones and usage of V(D)J genes A, UMAP of T cells derived from PBMCs. Clusters are shown in different colors (left) and TCR detection (right). **B**, The distribution of clone status of T cells in each condition. Clonal represents clonotype counts ≥2, no clonal represents clonotype counts=1, non-VDJ means cells without TCR. **C**, Bar plots showing the clonotype proportion in each condition. Conditions are shown in different colors. **D**, Bar plots showing the percentage of TCR in each T cell cluster. **E**, The association between the number of T cell clones and the number of cells per clonotype. The dashed line separates nonclonal and clonal cells. LOESS fitting is labeled as the solid line showing negative correlation between the two axes. **F**, Bar plots showing the clonotype proportion in each sample. Samples are shown in different colors. **G**, The distribution of clone status of T cell subsets across four conditions. **H**, Pie plots showing the clonal status percentage of T cells (left) and TCR clone differences across the Control, E-NoSEP, Y-SEP, E-SEP conditions. **I**, The top ten CDR3 usages are shown. Each bar is colored by condition identity

Although it is widely accepted that clonal expansion and usage of V(D)J genes in T cells continuously change with age or infection status [9], alterations in the TCR repertoire in elderly sepsis patients have not yet been studied at the single-cell level. We used scTCR-seq to explore the TCR repertoire across the four groups (Fig. 5 and Fig. S4). We observed that the distribution of clonotypes was lowest in the Control, which indicated that clonal expansion was obvious in the E-NoSEP, E-SEP, and Y-SEP groups (Fig. 5B, H). By analyzing the distribution of clone status among T cell subsets, we found that CD8 + T EM and CD8 + T EX showed high proportions (Fig. 5G). Notably, the CD8 + T EM subset showed the highest proportion of clonal cells in E-SEPs, whereas CD8 + T EX showed the highest proportion in Y-SEPs (Fig. 5G). The TCR clones differed across the four groups. Relative to the Control group, the other three groups showed a substantial decrease in unique clonotypes. Furthermore, the ratio of large clonal expansions (clonal size > 100) was higher in the E-SEP group (Fig. 5H), suggesting that TCR clonality increases with both sepsis and aging. We compared the usage of V(D)J genes across the four groups to explore the dynamics and gene preferences of TCRs induced by both sepsis and aging. First, we found that the usages of the top 10 complementarity-determining region 3 (CDR3) sequences differed across the four groups and were lower in the Control group (Fig. 5I). Next, we found that the usage of V(D)J genes also differed (Fig. S4). By comparing Y-SEP with Control, we observed that TRAV17 was downregulated, whereas TRAV2 was upregulated. In addition, TRAV27 was over-represented, while TRAV8-1 and TRAJ37 were down-regulated in the E-SEP group compared to the Y-SEP group, which might indicate abnormalities in TCR repertoires in elderly patients with sepsis. Taken together, these data showed increased clonality in T cells experiencing both aging and sepsis.

### Characterizing the differentiation trajectory of CD4 + T cell and CD8 + T cell in the elderly with sepsis 

**Fig. 6:**
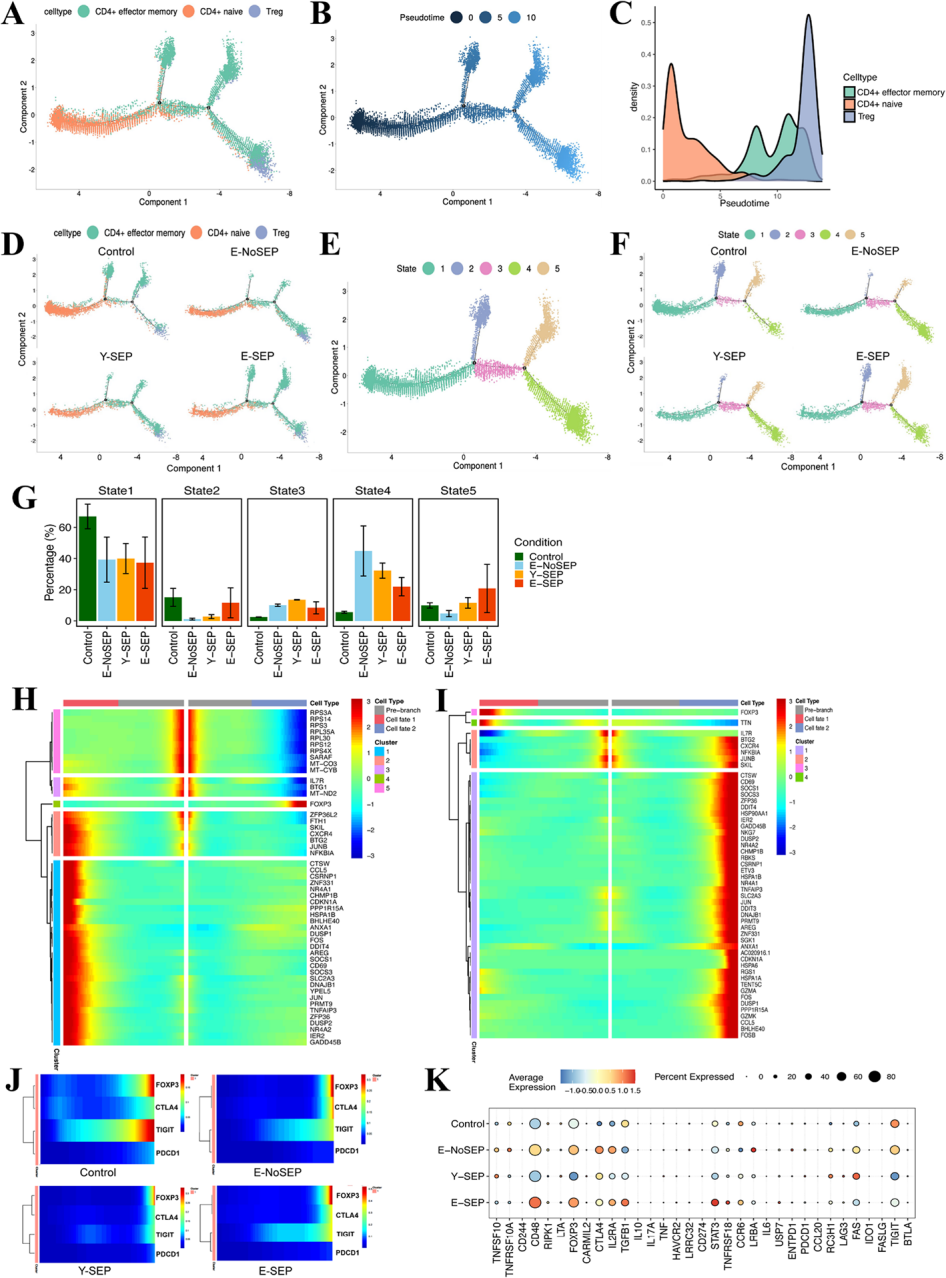
Trajectory and pseudotime analysis of CD4+T cells and expression of functional genes in Tregs. **A**, Trajectory analysis of CD4+T cell subsets color-coded by annotated cell subsets. **B**, Trajectory color-coded by pseudotime. **C**, Pseudotime analysis of CD4+T subsets color-coded by cell type. **D**, Trajectories of CD4+T cells across four conditions color-coded by cell type. **E**, Trajectory color-coded by cell state. The pseudotime trajectory showed 5 different states of CD4+T cells. **F**, Trajectories of CD4+T cells across four conditions color-coded by cell state. **G**, Proportions of 5 cell states in four conditions. Conditions are shown in different colors. **H**, Heatmap showing the clustering and expression kinetics of top 50 DEGs in branch 1. **I**, Heatmap showing the clustering and expression kinetics of top 50 DEGs in branch 2. **J**, Relative expression kinetics of *FOXP3*, *TIGIT*, *CTLA4*, and *PDCD1* over pseudo-time across four conditions. **K**, Dot plots showing the scaled expression of key functional genes, such as *FOXP3, TIGIT, TNFRSF18, *and* IL2RA,* in Tregs across four conditions. The color key from blue to red indicates low to high expression levels. The dot size indicates the percentage of cells that expressed genes

Since T cell subsets experienced significant cell composition and phenotypic alterations in elderly patients with sepsis, we further performed trajectory and pseudotime analyses to study the differentiation of CD4 + and CD8 + T cells via Monocle (Fig. 6 and Fig. S5). In the trajectory of CD4 + T cells, naïve cells were the initial point of the trajectory, transitioned to effector memory cells, and finally ended as Tregs (Fig. 6A-B). Based on the trajectory analysis across the four groups, the proportion of naïve CD4 + T cells was higher in the Control group than in the other three groups. In contrast, the proportions of CD4 + T EM and Tregs increased in both the Y-SEP and E-SEP groups (Fig. 6A-G). The entire trajectory was divided into five cell states based on branch points 1 and 2 according to the developmental order (Fig. 6B, E-G). In detail, state one CD4 + T cells differentiated into those characterized by states 2 and 3 and finally moved towards states 4 and 5. Furthermore, we investigated the branched expression of DEGs to characterize the alterations in relative expression over pseudo-time for distinct transcriptional states. The expression kinetics of the top 50 representative DEGs in branches 1 and 2 of the pseudo-time trajectory are shown in heatmaps (Fig. 6H-I). Focusing on *FOXP3*, we observed a general increased expression in cells in states 3 and 4, indicating differentiation of Tregs. In addition, the expression of inflammation- and activation-associated genes such as *CD69*, *CCL5*, *FOS*, *DDIT4*, *DUSP1/2*, and *JUN*, as well as the senescence hallmark gene *CDKN1A,* was upregulated in states 2 and 5 (Fig. 6H-I). Next, we examined the expression of several immune transcription factors of CD4 + T cells, while focusing on *FOXP3*, *TIGIT*, *CTLA4*, and *PDCD1* (Fig. 6J). We found that the trends in *FOXP3, CTLA4* and *TIGIT* expression were similar among the four groups and increased with Treg differentiation. To further comprehensively elucidate the alterations in Tregs, we compared the expression of functional genes across the four groups (Fig. 6K). Notably, we observed that the expression of *FOXP3, TIGIT, TNFRSF18,* and *IL2RA,* which are key functional genes of Tregs, increased significantly in E-SEP compared to Y-SEP. This suggested that the immunosuppressive effect of Tregs was enhanced in elderly patients with sepsis.

The trajectory and pseudotime analyses of CD8 + T cells are shown in Fig. S5. By branched expression analysis, we observed that the expression of several genes increased in both CD8 + T EFF and CD8 + T EM compared to that in naïve CD8 + T cells, including inflammation-associated genes (*DDIT4*, *DUSP1/2*, *JUN*, *JUNB*, *FOS*, and *CCL5*), cytotoxicity-associated genes (*GZMB*, *GZMA*, and *GNLY*), and KIR family genes (*KIR3DL1*, *KIRC2*, *KIR3DL2*, *KIRC3*, and *KIR2DL3*) (Fig. S5F-G).

### Alterations of CD4 + T and CD8 + T cell subsets in both inflammation- and mitochondria-associated pathways in elderly patients with sepsis 

**Fig. 7:**
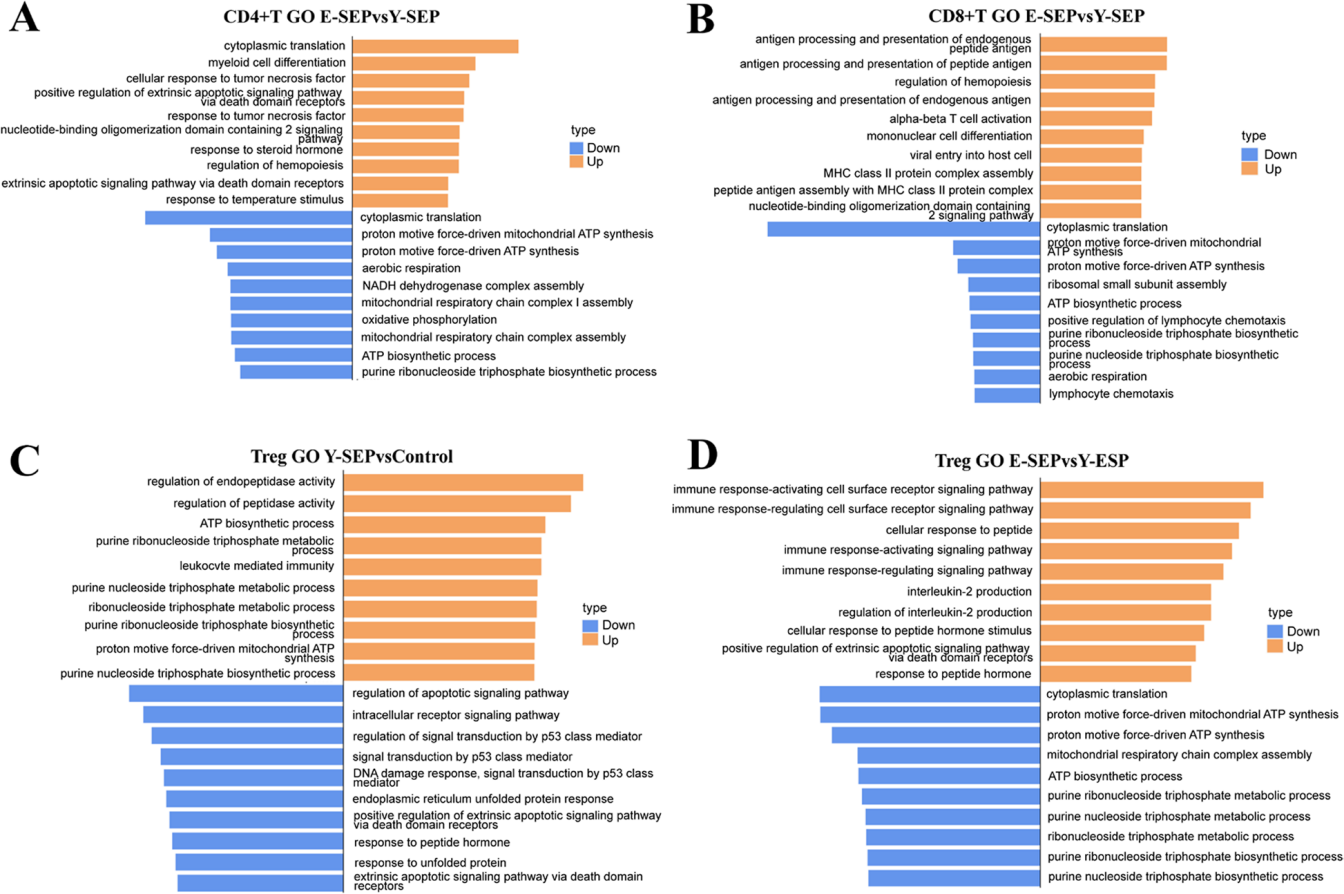
Enrichment of GO pathways in T cell subsets. **A, **Representative GO-BP terms enriched in the top 100 upregulated or downregulated DEGs based on functional enrichment analysis in total CD4+T cells between E-SEP and Y-SEP. GO terms are labeled with name, and sorted by −log_10_ (*P*) value. The top 10 enriched GO terms are shown. **B,** The GO enrichment analysis similar to **A**, but for total CD8+T cells. **C,** Representative GO-BP terms enriched in the top 100 upregulated or downregulated DEGs based on functional enrichment analysis in Tregs between Y-SEP and Control. GO terms are labeled with name, and sorted by −log_10_ (*P*) value. The top 10 enriched GO terms are shown. **D,** The GO enrichment analysis similar to **A**, but for Tregs

To evaluate the distinctive biological properties of the T cell subsets, we performed GO and KEGG enrichment analyses of DEGs between the Y-SEP and Control groups, and between the E-SEP and Y-SEP groups. For the total CD4 + T cells, we observed that interferon-γ signal, JAK-STAT signal, and apoptosis pathways were enriched in Y-SEP compared to Control (data not shown), which were in accordance with previously published studies by Venet et al. [20]. By comparing the DEGs of total CD4 + T cells from E-SEP and Y-SEP, both cellular responses to TNF and apoptosis were enriched, whereas mitochondria-associated GO pathways, including ATP synthesis, mitochondrial respiratory chain, and oxidative phosphorylation, were significantly inhibited (Fig. 7A). Consistently, KEGG enrichment showed that MAPK signaling, TNF signaling, cell senescence, and apoptosis pathways were upregulated, whereas ribosome and oxidative phosphorylation pathways were downregulated in E-SEP compared to Y-SEP (Fig. S6A). In contrast, we observed that mitochondria-associated pathways were enriched, while the apoptosis pathway was decreased in Tregs in Y-SEP compared to those in the Control (Fig. 7C), which was in line with the increased percentage and amplified suppressive effects of Tregs in sepsis previously reported by us [16]. Nevertheless, in contrast to Tregs from E-SEP versus Y-SEP, mitochondria-associated pathways decreased, whereas the apoptotic pathway increased (Fig. 7D).

Similarly, for total CD8 + T cells, we observed that mitochondria-associated pathways were inhibited, while inflammation-associated pathways (MAPK, IL-17, and TNF signaling) were increased in E-SEP compared to Y-SEP (Fig. 7B, S6B). Considering the significantly increased number of CD8 + T EX subsets shown in Fig. 4E, we performed a KEGG enrichment analysis. We found that MAPK signaling, TNF signaling, and apoptosis pathways were decreased in Y-SEP compared to those in the Control (Fig. S6D), which suggested an attenuated inflammatory response of CD8 + T EX in Y-SEPs and provided further clues to profound immune exhaustion.

Taken together, these data show that mitochondria-associated pathways were inhibited, while inflammation-associated pathways were upregulated in E-SEP compared to Y-SEP.

### Changes of metabolism-associated pathways in T cells in elderly patients with sepsis 

**Fig. 8:**
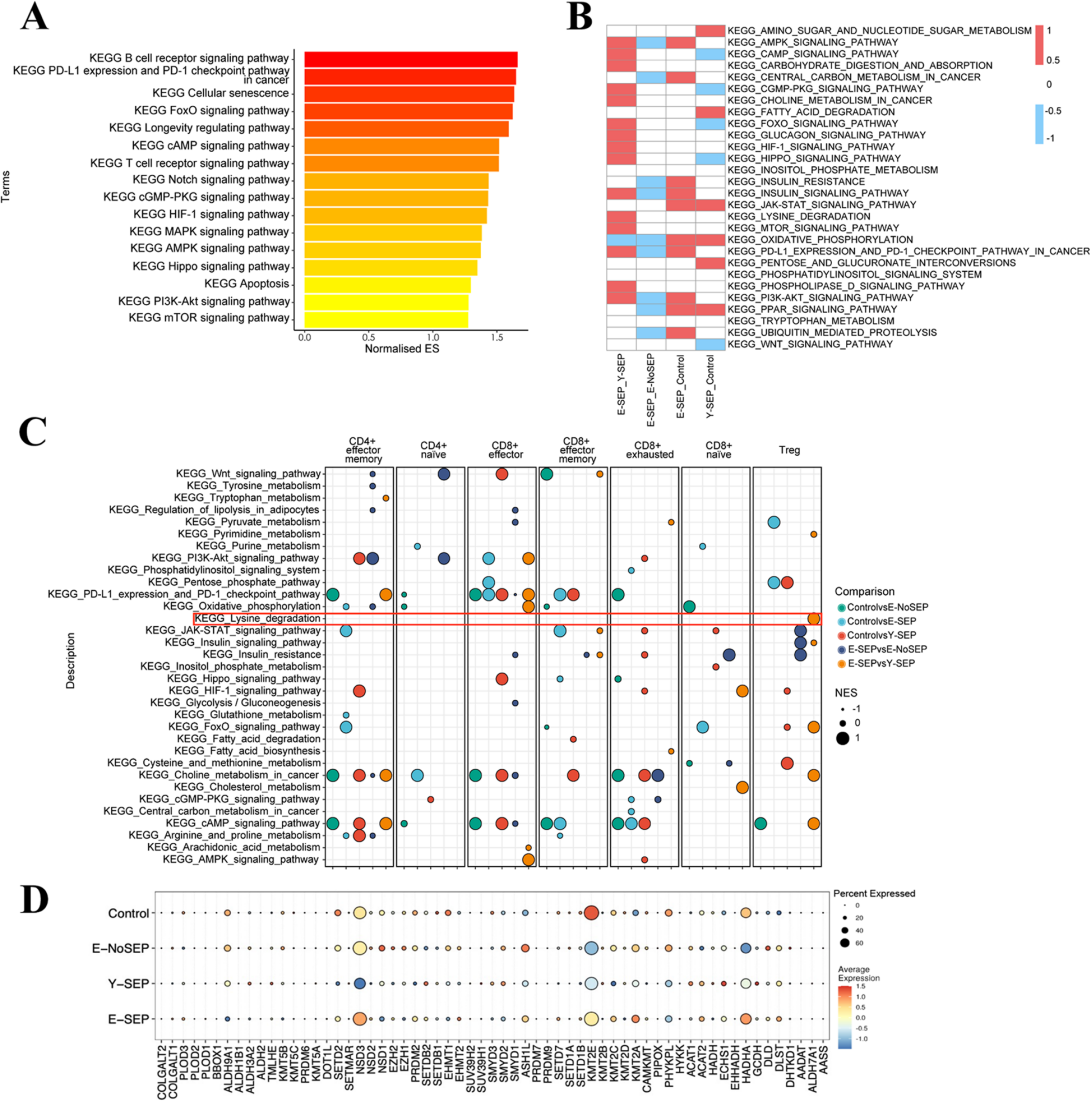
Metabolism-associated pathways enriched of T cells in the elderly with sepsis **A**, The GSEA enrichment plot showing the selected enriched genesets in KEGG pathway of T cells in E-SEP versus Y-SEP groups. And color-coded by normalized enrichment score. **B**, The GSEA NES heatmap of the enriched metabolism-associated genesets in KEGG pathway of T cells in E-SEP versus Y-SEP, E-SEP versus E-NoSEP, E-SEP versus Control, and Y-SEP versus Control. Red represents upregulated in the former group, and blue represents downregulated in the former group, respectively. **C**, Bubble plots showing the enriched metabolism-associated genesets in KEGG pathway of seven T cell subsets. Comparisons between two different conditions are shown in different colors, while orange indicates the comparison between E-SEP and Y-SEP. The dot size represents normalized enrichment score. **D**, Dot plots showing the expression of genes that involved in the lysine degradation pathway across four conditions. The color key from blue to red indicates low to high expression levels. The dot size indicates the percentage of cells that expresses genes

Given the significant changes of mitochondria-associated pathways in T cell subsets, we conducted Gene Set Enrichment Analysis (GSEA) of T cells to further analyze metabolism-associated pathways especially between E-SEP and Y-SEP. Notably, the enriched pathways of T cells in E-SEPs were quite different from those in Y-SEPs. We observed that several classic signaling pathways related to sepsis, such as the MAPK, PI3K-Akt, and apoptosis pathways, were enriched in E-SEP compared to Y-SEP (Fig. 8A). Additionally, the cellular senescence pathway, PD-L1 expression and PD-1 checkpoint, and FoxO signaling pathways were enhanced in the E-SEP group (Fig. 8A). Remarkably, metabolism-associated pathways, including mTOR, cAMP, AMPK, HIF-1, and cGMP-PKG signaling, were enriched in E-SEP (Fig. 8A), suggesting that alterations in metabolism might be essential for disease progression in elderly sepsis patients. We explored the enriched metabolism-associated genesets across the four groups in a global landscape (Fig. 8B). Notably, we observed that the T cells of elderly patients with sepsis showed much more upregulated metabolism-associated pathways, such as AMPK signaling, carbohydrate digestion and absorption, and mTOR signaling pathways (Fig. 8B), indicating that immunometabolism is an important feature underlying the pathophysiology of sepsis and aging. To explore whether alterations in metabolism-associated pathways were driven by certain cell subtypes, we further expanded the pathway enrichment analysis to each T cell subset. The differences in metabolism-associated pathways between different T cell subsets are shown in Fig. 8C. We observed that metabolism-associated pathways were enriched in Tregs in E-SEPs compared with those in Y-SEPs. Surprisingly, the lysine degradation pathway was enriched only in the Treg subset in E-SEP compared to that in Y-SEP (Fig. 8C). Moreover, the genes involved in the lysine degradation pathway, such as *NSD3, HADHA*, and *KMT2A*, were upregulated in the E-SEP group (Fig. 8D). Coupled with the enhanced immunosuppressive effect of Tregs, as described above, lysine degradation may contribute to the disturbed immunometabolism mediated by Tregs in elderly patients with sepsis.

### Potential cell–cell interactions between immune cells in elderly sepsis patients

**Fig. 9:**
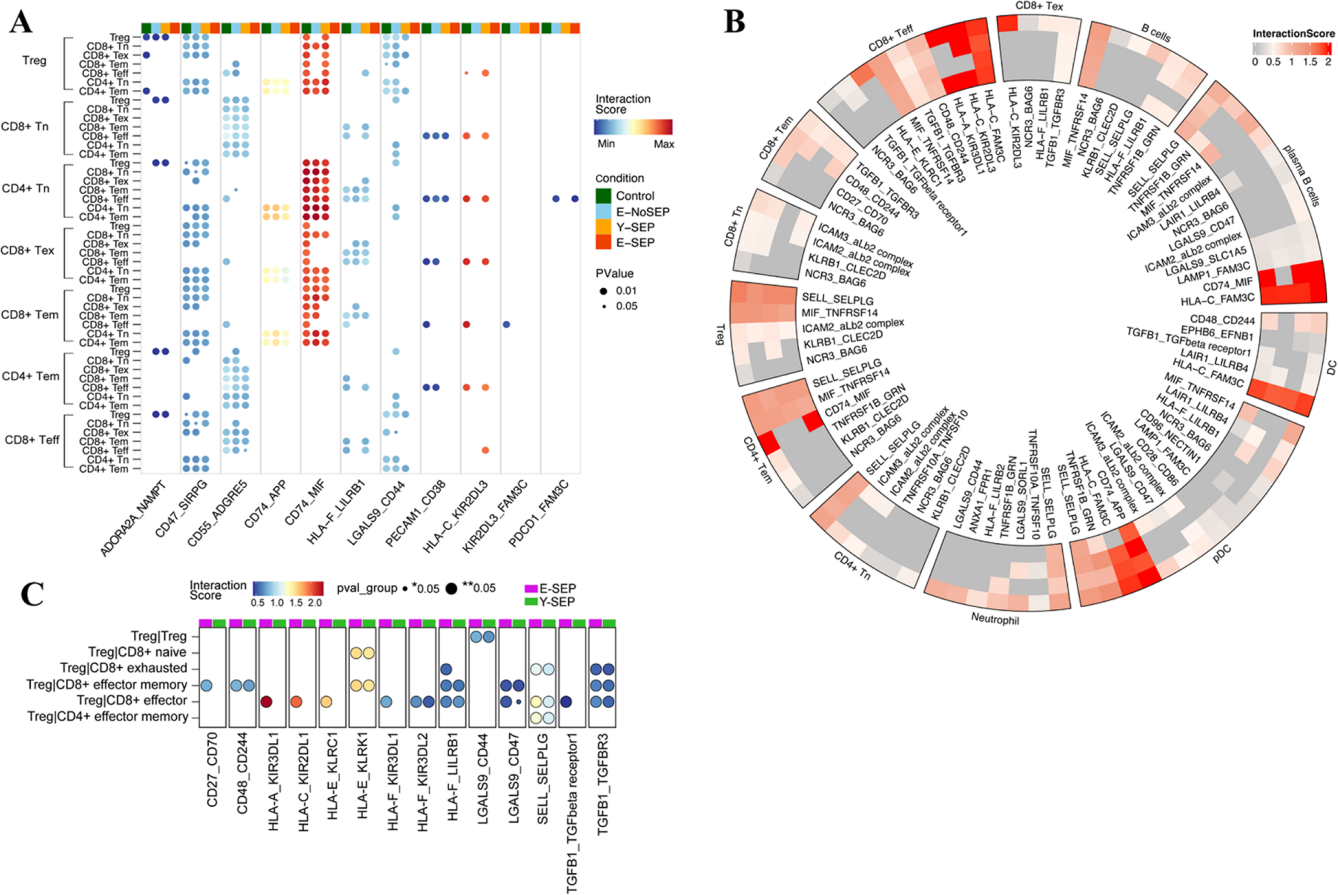
Potential cell–cell interactions between immune cells **A**, Bubble plots showing the mean interaction strength for selected ligand–receptor pairs between immune cell clusters across four conditions. The dot size indicates *P* value generated by permutation test, colored by interaction strength levels. The former cells provide receptors (the former: receptor; the latter: ligand). **B**, The heatmap showing cell-cell interaction analysis of ligand-receptor pairs which upregulates in E-SEP between Tregs and other immune cell subsets using cellphoneDB. Ligands of naïve CD8+T, CD8+T EX, CD8+T EM, CD8+T EFF, naïve CD4+T, CD4+T EM, B cells, plasma B cells, Tregs, pDCs, neutrophils, and cDCs. And Tregs are cells providing receptors (the former: receptor; the latter: ligand), colored by interaction strength levels. The circle from the inside out represents Control, E-NoSEP, Y-SEP, E-SEP, respectively. **C**, Bubble plots showing the mean interaction strength for selected ligand–receptor pairs between Tregs and various immune cell clusters in E-SEP and Y-SEP. The dot size indicates *P* value generated by permutation test, colored by interaction strength levels. Tregs are cells providing receptors (the former: receptor; the latter: ligand)

Next, we investigated the cellular interactions between immune cell subtypes using CellphoneDB (Fig. 9). Within the T-cell subsets, we observed several ligand-receptor pairs that were not expressed in the E-SEP group, including ADORA2A-NAMPT, CD47-SIRPG, CD55-ADGRE5, CD74-MIF, HLA-F-LILRB1, LGALS9-CD44, and PECAM1-CD38 (Fig. 9A). The impaired interaction of these ligand-receptor pairs is closely related to dysregulated inflammatory responses, contributing to aggravated immune dysfunction in elderly individuals with sepsis [21–26]. In addition, the HLA-C-KIR2DL3 and KIR2DL3-FAM3C pairs were expressed only in the Control and Y-SEP groups (Fig. 9A), which may be associated with aging. Concurrently, PDCD1-FAM3C, which plays an essential role in immunosuppression, as previously reported [27], was expressed only in the aging groups (including E-SEP and E-NoSEP) (Fig. 9A).

In elderly patients with sepsis, Tregs exhibit enhanced immunosuppressive effects, which are usually exerted by interacting with other cells. Thus, we further analyzed the cellular interactions between Tregs and other immune cell subsets to identify the target cells of Tregs. Enhanced interactions of the ligand-receptor pairs in the E-SEP group are shown in Fig. 9B. Notably, CD8 + T cells appeared to be the closest cooperators of Tregs, suggesting that Tregs exert their regulatory function on CD8 + T cells during disease progression in elderly sepsis patients (Fig. 9B). We compared the strengths of the ligand–receptor pairs contributing to Treg-CD8 + T cell interactions between E-SEP and Y-SEP. We found that several ligand-receptor pairs involving in the immunosuppressive function of Tregs, including TGFB1-TGFBR3, TGFB1-TGFβ receptor1, CD48-CD244, CD27-CD70, and SELL-SELPLG, showed higher interaction potentials in E-SEP (Fig. 9C). In addition, it has recently been reported that both LGALS9 and CD47 are co-inhibitory molecules, suggesting the LGALS9-CD47 pair might also contribute to the enhanced immunosuppressive effect of Tregs in elderly patients with sepsis. Interestingly, for the interactions between Tregs and CD8 + T cells, ligand-receptor interactions were concentrated mainly on HLA and KIR family, including HLA-A-KIR3DL1, HLA-C-KIR2DL1, HLA-E-KLRK1, HLA-F-KIR3DL1, and HLA-F-KIR3DL2 (Fig. 9C). Remarkably, among these HLA-KIR pairs, HLA was expressed on Tregs, while KIR was expressed on CD8 + T cells, which has not yet been reported. Interactions of HLA-KIR pairs between Tregs and CD8 + T cells may play a critical role in immune dysfunction in elderly sepsis patients.

## Discussion

Sepsis is considered a dysregulated host responses to severe infections [1]; however, the underlying immunopathogenic mechanisms remain unclear. Overactive immune responses and immunosuppression or immune exhaustion can directly and/or indirectly contribute to poor prognosis in patients [5]. Elderly patients, a special population influenced by immunosenescence, are more susceptible to sepsis and have a worse prognosis as compared to young patients [6]. Several previous studies have reported the characteristics of immune features in sepsis and aging respectively, which have helped us understand the potential immunopathologies of these two aspects. However, a comprehensive landscape of the cellular and molecular immunological features in elderly patients with sepsis remains lacking. To address this issue, we performed scRNA-seq and scTCR-seq to explore the immunological landscape in elderly sepsis patients at single-cell resolution and elucidate the alterations in immune cell composition, expression heterogeneities, and cellular interactions. This will facilitate a better understanding of the critical nodes between the dysregulated immune system and severe infections that occurs in elderly patients.

Our study provides a comprehensive and integrated immune landscape of elderly patients with sepsis at the single-cell level. Here, we present an unbiased visualization of the immunological hallmarks. First, immune cells showed significantly and concertedly increased apoptosis in elderly sepsis patients, as evidenced by enrichment in the apoptosis signaling pathway and increased apoptosis scores in the majority of immune cell subpopulations in E-SEP. Second, enhanced immune exhaustion has been observed in elderly sepsis patients. Functional enrichment analysis revealed that the ribosome pathway was significantly inhibited in almost all immune cell subtypes in E-SEP compared to that in Y-SEP; the downregulation of which has been reported to be closely related to exhaustion [28]. In addition, the abundance of both exhausted CD8 + T and Treg subsets increased and exhaustion scores were elevated in elderly patients with sepsis. Third, an overactive inflammatory state was observed in elderly sepsis patients, and was characterized by the upregulation of inflammation-associated genes and the enrichment of inflammatory pathways. Remarkably, we identified upregulated genes of both sepsis and aging groups were involved in interferon-γ-associated signals, which indicated that inflammaging was amplified in elderly patients with sepsis. Fourth, the proportion of Tregs is significantly higher in elderly sepsis patients. In addition, the expression of *FOXP3, CTLA4, TIGIT, TNFRSF18,* and *IL2RA,* key functional genes of Tregs, was increased in the E-SEP group, suggesting that the immunosuppressive effect of Tregs was enhanced in the elderly with sepsis. Fifth, metabolism-associated pathways were upregulated in the T cells of elderly patients with sepsis, whereas mitochondria-associated pathways were significantly inhibited. Given these metabolic alterations, immunometabolism, including mitochondrial pathways, may be an important characteristic underlying the pathophysiology of sepsis and aging. Surprisingly, we found that the lysine degradation pathway was upregulated only in the Treg subset, and the genes involved in this pathway were increased in E-SEPs, suggesting that lysine supplementation might be an effective therapeutic method for elderly sepsis patients. Finally, cell–cell interaction analysis showed that the expression profile of ligand-receptor pairs was probably associated with aggravated immune dysfunction in elderly patients with sepsis. For the interactions between Tregs and CD8 + T cells, ligand-receptor interactions were concentrated mainly on HLA and KIR family, which have not yet been published.

Notably, innate immune cells not only showed changes in cell composition but also exhibited significant phenotype shifts and functional alterations in elderly patients with sepsis. Both monocytes and DCs exhibited decreased antigen-presenting ability and turned to an overactive inflammatory and aging phenotype in elderly patients with sepsis. This was evidenced by downregulated *TXNIP* and *HLA-DQA2* gene expression and upregulated inflammation- and senescence-associated gene expression, as well as alterations in signaling pathways in E-SEP. For neutrophils, we observed that neutrophil extracellular trap formation, complement and coagulation cascades, and platelet activation pathways were concertedly and strikingly enhanced in E-SEP, which might be closely related to the aggravation of microcirculation damage in elderly sepsis patients and worsen their prognosis.

Moreover, alterations in adaptive immune cells should not be ignored. Our data showed that T cells in elderly patients with sepsis exhibited an immune phenotype shifts into effector, memory, and exhausted populations. This was indicated by the abundance of exhausted CD8 + T and Treg subsets, decreased proportion of naïve T cell subsets, and increased active-state T cell subtypes coupled with the differentiation of CD4 + T and CD8 + T cells using trajectory analysis and elevated cytotoxicity and exhaustion scores. In addition, we observed increased clonality in T cells experiencing both aging and sepsis combined with different immunodominant epitopes, elucidating the abnormal immune states induced by both aging and sepsis. Functional enrichment analysis of T cells revealed that classic signaling pathways related to sepsis were enriched in E-SEP compared to Y-SEP, indicating that the characteristics of sepsis were much more prominent in elderly sepsis patients, which might be due to the synergistic effects of both aging and sepsis. Notably, we identified strong interferon-γ responses in both aging and sepsis groups, which may play an essential role in aggravated sepsis and senescence features and could be a therapeutic target for elderly sepsis patients.

The immunopathogenesis of disease progression in elderly patients with sepsis is characterized by broad immune cell apoptosis, profound immune exhaustion, amplified immunosuppressive effects of Tregs, deranged inflammaging, and dysfunction of immunometabolism, including mitochondrial metabolism. Remarkably, previous studies have shown that IL-7 administration can reverse sepsis-induced lymphopenia by inhibiting apoptosis and improving the proliferation of T lymphocytes, which was recently demonstrated in a clinical trial [29, 30]. In addition, IL-7 increases the activation and promotes the rejuvenation of T cells in patients with immunosenescence, as reported by Marton et al. [31]. Considering both aggravated immune cell apoptosis and profound immune exhaustion in elderly patients, with sepsis, IL-7 may be an underlying immunotherapeutic strategy, and its efficacy is highly anticipated. Since we observed that the antigen-presenting ability of both monocytes and DCs was significantly reduced in elderly patients with sepsis, GM-CSF could be a therapeutic alternative to improve innate immune dysfunction, which shows promising utility for attenuating immunoparalysis in sepsis [32]. In addition, the key role of mitochondria in the pathogenesis of sepsis is being increasingly recognized [33], which worsens immune dysfunction mainly by increasing ROS production, reducing ATP generation, and triggering the intrinsic pathway of apoptosis [16, 33]. We identified a striking dysfunction in the mitochondrial metabolism in elderly patients with sepsis, which represents an attractive new therapeutic target for this disease. Although the mechanisms underlying immunometabolic dysfunction appear to be complex, alterations in metabolic processes may play essential roles in immune dysfunction of elderly sepsis patients. Notably, the lysine degradation pathway was upregulated only in the Treg subset. Coupled with the enhanced immunosuppressive effect of Tregs, lysine degradation may contribute to disturbed immunometabolism mediated by Tregs in elderly patients with sepsis. Combined with the benefits of lysine supplementation in both aging and sepsis patients [34, 35], lysine might be an effective treatment for elderly patients with sepsis; however, considerable experiments are required to verify this hypothesis and further elucidate the underlying mechanisms.

However, our study also had a few limitations. First, the sample size was relatively small. Future studies with larger sample sizes may be helpful in determining the characteristics of the dysregulated immune system and further recognizing the extraordinarily complicated regulatory networks between immune cells and molecules in elderly patients with sepsis. In addition, the expression profile of ligand-receptor pairs, especially HLA-KIR between Treg and CD8 + T cell interactions, has not been validated experimentally because of the difficulty in isolating sufficient immune cell subsets and co-culturing them to detect cell–cell interactions. Moreover, since we observed striking alterations in immune cell metabolic processes contributing to immune dysfunction in elderly patients with sepsis, future studies focusing on metabolomics are urgently needed to comprehensively decipher the mechanisms of immunometabolism dysfunction.

Collectively, our study explored immunological alterations in elderly patients with sepsis at the single-cell level, including immune cell composition, expression heterogeneities, and cellular interactions, which provided a novel and comprehensive understanding of the underlying mechanisms of the dysregulated immune system. Furthermore, the integrated description of immunological hallmarks in our study lays the foundation for future characterization of the complex cellular and molecular immunological features, and sheds light on new immunotherapy strategies as well as improved prognosis in elderly sepsis patients.

### Supplementary Information

Supplementary Material 1.

Supplementary Material 2.

## Data Availability

The raw sequence data reported in this paper have been deposited in the Genome Sequence Archive (Genomics, Proteomics & Bioinformatics 2021) in National Genomics Data Center (Nucleic Acids Res 2022), China National Center for Bioinformation / Beijing Institute of Genomics, Chinese Academy of Sciences (GSA-Human: HRA007926) that are publicly accessible at https://ngdc.cncb.ac.cn/gsa-human.
