# Correction: Single-cell landscape of immunological responses in elderly patients with sepsis

**DOI:** 10.1186/s12979-024-00456-x

**Published:** 2024-08-02

**Authors:** Wanxue He, Chen Yao, Kaifei Wang, Zhimei Duan, Shuo Wang, Lixin Xie

**Affiliations:** 1 Department of Pulmonary and Critical Care Medicine, Xuanwu Hospital Capital Medical University, Beijing, China; 2 College of Pulmonary and Critical Care Medicine, The Eighth Medical Center, Chinese PLA General Hospital, Beijing, China; 3 CAS Key Laboratory of Pathogen Microbiology and Immunology, Institute of Microbiology, Chinese Academy of Sciences, Beijing, China

**Correction: Immun Ageing 21**,** 40 (2024)**

10.1186/s12979-024-00446-z

Following publication of the original article [1], the authors reported an error in this article. The correct **Availability of data and materials** should be “The raw sequence data reported in this paper have been deposited in the Genome Sequence Archive (Genomics, Proteomics & Bioinformatics 2021) in National Genomics Data Center (Nucleic Acids Res 2022), China National Center for Bioinformation / Beijing Institute of Genomics, Chinese Academy of Sciences (GSA-Human: HRA007926) that are publicly accessible at https://ngdc.cncb.ac.cn/gsa-human.”

## References

[1] He W, Yao C, Wang K, et al. Single-cell landscape of immunological responses in elderly patients with sepsis. Immun Ageing. 2024;21:40. 10.1186/s12979-024-00446-z. 38909272 10.1186/s12979-024-00446-z PMC11193269

