# Supplementary material for: Single-cell landscape of immunological responses in elderly patients with sepsis
Source: Immun Ageing. 2024 Jun 22;21:40. doi: 10.1186/s12979-024-00446-z (PMC11193269; doi:10.1186/s12979-024-00446-z)
Supplement: Supplementary file 2 — Supplementary Material 2. [file 12979_2024_446_MOESM2_ESM.docx]

**Extended Data Figure 1**: Cellular populations and hallmark genes identified.

**A**, Visualization of all indentified cell types. The UMAP embedding single cells from Control (n=3), E-NoSEP (n=3), Y-SEP (n=3), E-SEP (n=3) samples, showing the formation of 14 clusters (cells for CD4+T, CD8+T, γδT, monocyte, macrophage, DC, pDC, neutrophil, B, plasma B, erythroid, cycling cell, platelet, and stress cell, respectively). Each dot corresponds to a single cell, colored according to cell type.

**B**, Dot plots showing the expression of hallmark genes for each cell cluster and cell type. Color scale corresponds to z-score, the color key from blue to purple indicated low to high expression levels. The dot size indicated the percentage of cells that expressed genes.

**C**, The UMAP projection of each studied individual, including 3 Control (S1 to S3), 3 E-NoSEP (S7 to S9), and 3 Y-SEP (S10 to S12), 3 E-SEP (S4, S6, and S13). Each dot corresponds to a single cell, colored according to cell type.

**Extended Data Figure 2:** Transcriptional profiling of innate immune cell subsets in E- SEP and Y-SEP patients.

**A**, Volcano plot showing DEGs in DCs between E-SEP and Y-SEP. The green dots indicate down-regulated DEGs while the red dots indicate up-regulated DEGs. Example genes are labeled with gene name. Statistically significant DEGs were identified with Benjamini–Hochberg adjusted *P* value (two-sided unpaired Mann–Whitney *U*-test) ≤0.01 and average log_2_ fold change ≥0.25.

**B**, Volcano plot similar to **A**, but for monocytes.

**C**, Volcano plot similar to **A**, but for pDCs.

**D**, The KEGG pathway enrichment analysis of the top 100 upregulated DEGs in pDCs between E-SEP and Y-SEP. Pathways are labeled with name, and sorted by −log_10_ (*P*) value. A darker color indicates a smaller *P* value.

**E**, The KEGG pathway enrichment analysis of the top 100 downregulated DEGs in pDCs between E-SEP and Y-SEP. Pathways are labeled with name, and sorted by −log_10_ (*P*) value. A darker color indicates a smaller *P* value.

**F**, Volcano plot similar to **A**, but for macrophages.

**G**, The KEGG pathway enrichment analysis similar to **D**, but for macrophages.

**H**, The KEGG pathway enrichment analysis similar to **E**, but for macrophages.

**I**, Volcano plot similar to **A**, but for neutrophils.

**J**, The KEGG pathway enrichment analysis of the top 100 upregulated DEGs in neutrophils between E-SEP and Y-SEP. Pathways are labeled with name, and sorted by −log_10_ (*P*) value. A darker color indicates a smaller *P* value.

**K**, Box plots of the apoptosis score of neutrophils across different conditions. Conditions are shown in different colors. Horizontal lines represent median values, with whiskers extending to the furthest data point within a maximum of 1.5 × interquartile range. All differences with *P* < 0.05 are indicated. *****P* < 0.0001; unpaired T-test was used for analysis.

**Extended Data Figure 3**: Cell compositions in individuals and transcriptomic profiles of T cells.

**A**, UMAP plots showing the expression of canonically cell markers used to label each cluster of T cells. Data are colored according to expression levels.

**B**, Dot plots showing the expression of hallmark genes for each subtype of T cells. Color scale corresponds to z-score, the color key from blue to purple indicates low to high expression levels. The dot size indicates the percentage of cells that expresses genes.

**C**, The UMAP plot of all cells in the study colored by four conditions.

**D**, The UMAP plot of all cells in the study colored by 12 individual samples.

**E**, The top dot plot shows the sum of absolute counts of lymphocytes and monocytes in the PBMCs of each sample. The bottom bar plot shows the relative contributions of each T cell subset in each studied subject.

**F**, Scatter-plot showing 73 DEGs in T cells of E-NoSEP (*n* = 3) or E-SEP patients (*n* = 3) in comparison with Control (*n* = 3). Each red dot represents an individual gene with Wilcox test adjusted *P* value ≤0.01 and average log2 fold change ≥0.25 in the E-NoSEP/Control and E-SEP/Control comparisons.

**G**, Representative GO-BP terms enriched in the DEGs which are colored in red as in **F**. A redder one indicates a bigger *P* value. The top 20 enriched GO terms are shown.

**Extended Data Figure 4: Biased expanded TCR clones and usage of V(D)J genes.**

Usage of TRAV (**A**), TRAJ (**B**), TRBV (**C**) and TRBJ (**D**) genes across different groups of patients. Conditions are shown in different colors. Error bars represent ± s.e.m.. All differences with *P* < 0.05 are indicated. **P* < 0.05; two-sided unpaired Mann-Whitney U test was used for analysis.

**Extended Data Figure** **5**: Trajectory and pseudotime analysis of CD8+T cells.

**A**, Trajectory analysis of CD8+T cell subsets color-coded by annotated cell subset.

**B**, Trajectory color-coded by pseudotime.

**C**, Trajectory color-coded by cell state. The pseudotime trajectory showed 5 different states of CD8+T cells.

**D**, Trajectories of CD8+T cells across four conditions color-coded by cell type.

**E**, Trajectories of CD8+T cells across four conditions color-coded by cell state.

**F**, Heatmap showing the clustering and expression kinetics of top 50 DEGs in branch 1.

**G**, Heatmap showing the clustering and expression kinetics of top 50 DEGs in branch 2.

**Extended Data Figure 6**: Enrichment analysis of pathways in T cell subsets.

**A**, The KEGG pathway enrichment analysis of the top 100 upregulated or downregulated DEGs in total CD4+T cells between E-SEP and Y-SEP. Selected pathways are labeled with name, and sorted by −log_10_ (*P*) value.

**B**, The KEGG pathway enrichment analysis similar to **A**, but for total CD8+T cells.

**C**, Representative GO-BP terms enriched in the top 100 upregulated or downregulated DEGs based on functional enrichment analysis in CD8+T EFF cells between E-SEP and Y-SEP. GO terms are labeled with name, and sorted by −log_10_ (*P*) value. The top 10 enriched GO terms are shown.

**D**, The KEGG pathway enrichment analysis of the top 100 downregulated DEGs in CD8+T EX cells between Y-SEP and Control. Selected pathways are labeled with name, and sorted by −log_10_ (*P*) value.
